# An HPV 16 L1-based chimeric human papilloma virus-like particles containing a string of epitopes produced in plants is able to elicit humoral and cytotoxic T-cell activity in mice

**DOI:** 10.1186/1743-422X-6-2

**Published:** 2009-01-06

**Authors:** Georgina Paz De la Rosa, Alberto Monroy-García, María de Lourdes Mora-García, Cristina Gehibie Reynaga Peña, Jorge Hernández-Montes, Benny Weiss-Steider, Miguel Angel Gómez Lim

**Affiliations:** 1 Centro de Investigación y de Estudios Avanzados (CINVESTAV), Unidad Irapuato, Km. 9.6 Libramiento Norte, 36500 Carretera Irapuato-León. Irapuato, Guanajuato, Mexico; 2 Laboratorio de Inmunobiología, Unidad de Investigación en Diferenciación Celular y Cáncer. FES-Zaragoza, UNAM, México; 3 Unidad de Investigación Médica en Enfermedades Oncológicas. IMSS, CMN SXXI, México

## Abstract

**Background:**

Even though two prophylactic vaccines against HPV are currently licensed, infections by the virus continue to be a major health problem mainly in developing countries. The cost of the vaccines limits wide-scale application in poor countries. A promising strategy for producing affordable and efficient vaccines involves the expression of recombinant immunogens in plants. Several HPV genes have been expressed in plants, including L1, which can self-assemble into virus-like particles. A plant-based, dual prophylactic/therapeutic vaccine remains an attractive possibility.

**Results:**

We sought to express in tomato plants chimeric HPV 16 VLPs containing L1 fused to a string of epitopes from HPV 16 E6 and E7 proteins. The L1 employed had been modified to eliminate a strong inhibitory region at the 5' end of the molecule to increase expression levels. Several tomato lines were obtained expressing either L1 alone or L1-E6/E7 from 0.05% to 0.1% of total soluble protein. Stable integration of the transgenes was verified by Southern blot. Northern and western blot revealed successful expression of the transgenes at the mRNA and protein level. The chimeric VLPs were able to assemble adequately in tomato cells. Intraperitoneal administration in mice was able to elicit both neutralizing antibodies against the viral particle and cytotoxic T-lymphocytes activity against the epitopes.

**Conclusion:**

In this work, we report for the first time the expression in plants of a chimeric particle containing the HPV 16 L1 sequence and a string of T-cell epitopes from HPV 16 E6 and E7 fused to the C-terminus. The particles were able to induce a significant antibody and cytotoxic T-lymphocytes response. Experiments *in vivo *are in progress to determine whether the chimeric particles are able to induce regression of disease and resolution of viral infection in mice. Chimeric particles of the type described in this work may potentially be the basis for developing prophylactic/therapeutic vaccines. The fact that they are produced in plants, may lower production costs considerably.

## Background

The human papillomaviruses (HPV) comprise a heterogeneous group of more than 130 epitheliotropic genotypes, 16 of which are considered "high-risk" types and linked with the development of malignant disease [1]. High-risk types, HPV 16 and HPV 18, are the main etiological agents of cervical cancer, with HPV 16 being involved in about half of all cases of cancer [2]. HPV are responsible for approximately half a million new cervical cancer cases every year and almost 250,000 deaths per year, most of them occurring in developing countries [2]. Among the various HPV components, the major structural protein of the capsid, L1, has been the antigen of choice for the development of prophylactic vaccines. L1 can self-assemble into virus-like particles (VLPs), which are very efficient in inducing an immune response. VLP-induced antibodies provide protective immunity against challenge with infectious viruses in animal models [3,4]. Two L1-based, HPV vaccines have been licensed in several countries after demonstration of an acceptable benefit/risk profile [5], one (Gardasil^®^) contains two high-risk (16 and 18) and two low-risk (6 and 11) types and the other (Cervarix^®^) is composed of two high-risk types, 16 and 18.

Parenteral vaccines, such as that for HPV, are expensive to produce and costly to administer. Thus, many diseases that are controlled effectively in industrialized countries are controlled poorly at best in poor countries. A promising strategy for producing affordable and efficient vaccines involves the expression of recombinant immunogens in plants. Plants provide a number of advantages for production of recombinant proteins over conventional systems including low cost, increased safety and scalable production [6]. They are a potential source of a wide range of biopharmaceuticals that is not dependent upon downstream processing technology to ensure protein folding, particle assembly and stability. In consequence, a plant-based biopharmaceutical expression system makes possible either testing of an oral therapy strategy by simply feeding with edible plant tissues or purification of the compound from the plant, which would reduce processing costs. A number of antigens from different origins have been expressed in various plants and HPV antigens have been no exception. Plants such as tobacco, potato, tomato and Arabidopsis have been employed for the production of human and animal papilloma L1 VLPs, which appeared similar in size and structure to authentic papilloma virus VLPs as shown by electron microscopy studies [7-14]. In addition, plant-produced VLPs were immunogenic when administered in mice either orally or intraperitonally [7,8,14].

On the other hand, since capsid proteins are not expressed at detectable levels by HPV-transformed cells, therapeutic vaccines generally target the nonstructural early viral antigens. The E6 and E7 oncogenic proteins, which are critical to the induction and maintenance of cellular transformation and co-express in the majority of HPV-containing carcinomas, are potential targets in the development of an immunotherapy against HPV infection [15,16]. Although other early viral antigens show promise for vaccination against papillomas, therapeutic vaccines targeting E6 and E7 may provide the best opportunity to control HPV-associated malignancies. These proteins have been demonstrated to contain several reactive T-cell epitopes which may play a role as tumor rejection antigens in humans [17]. Cell-mediated immunity responses to these epitopes correlates significantly with regression of disease and resolution of viral infection within 12 months [18,19]. Since HPV VLPs can be employed as vehicles to deliver epitopes or genes [20], we hypothesized that chimeric VLPs (cVLPs) containing L1 fused to T-cell epitopes from E6 and E7 might be able to elicit both neutralizing antibodies against the viral particle and cytotoxic T-lymphocytes (CTL) activity against the epitopes and induce regression of disease and loss of HPV infection. In this work, we report for the first time the expression of a chimeric particle containing the HPV 16 L1 sequence and a string of T-cell epitopes from HPV 16 E6 and E7 fused to the C-terminus. The particle was expressed in tomato cells and was able to stimulate significant antibody and cell-mediated immune responses in mice.

## Results

### Tomato plant transformation with LI-E6/E7 fusion gene constructs

We transformed tomato cotyledons with the construct LI-E6/E7-2300 or with L1 alone via *A. tumefaciens *infection. We employed the modified L1 cDNA reported by Collier et al [21], in which the strong inhibitory elements located in the first 514 nucleotides of the L1 gene were modified. A synthetic peptide containing three E7 and one E6 epitopes mediating cytotoxic activity [18,19,17] was fused to the C terminus of the optimized L1, which removed the last 34 amino acids at the 3' end. We recovered 9 plants for L1 and 5 for LI-E6/E7 on selective medium which were grown in the greenhouse to maturity and selfed. These plants were tested for NPTII expression. Their progeny was analyzed by Southern blot and employed for subsequent experiments. Of nine lines found to express L1 mRNA, only two presented a normal phenotype. The rest of the lines were not transferred to the greenhouse due to poor growth of tissue culture plantlets or because they showed stunted growth in the greenhouse and did not flower in time (data not shown). Of five lines expressing LI-E6/E7, we were able to recover only three.

### L1 expression at the transcriptional and translational level

**Figure 1:**
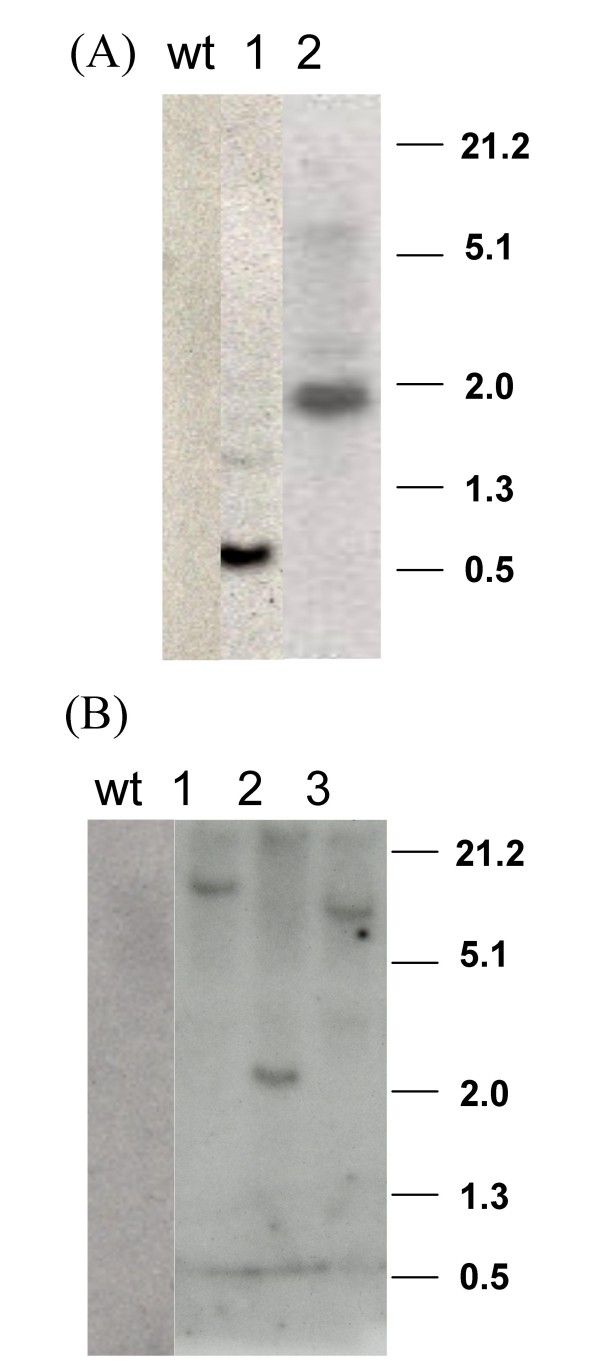
**L1 HPV-16 transgenes integration**. Twenty micrograms of genomic DNA were extracted from tomato leaves as described in the text and restricted with EcoRI, separated by agarose electrophoresis and blotted on to Hybond-N^+ ^nylon membrane. The membrane was hybridized with the 670 bp EcoRI/BamH1 fragment of the HPV-16 L1 gene. (A) Transgenic lines L1. (B) Transgenic lines L1-E6/E7. wt: non-transformed plant. Molecular weight markers are indicated on the right.

Integration of L1 or LI-E6/E7 fusion in the tomato lines was confirmed by Southern blot hybridization. We found that the two lines expressing L1 contained one copy whereas all three lines transformed with L1-E6/E7 contained also a single copy of the transgene (Fig. 1). DNA from wild type plants did not hybridize with the probe.

**Figure 2:**
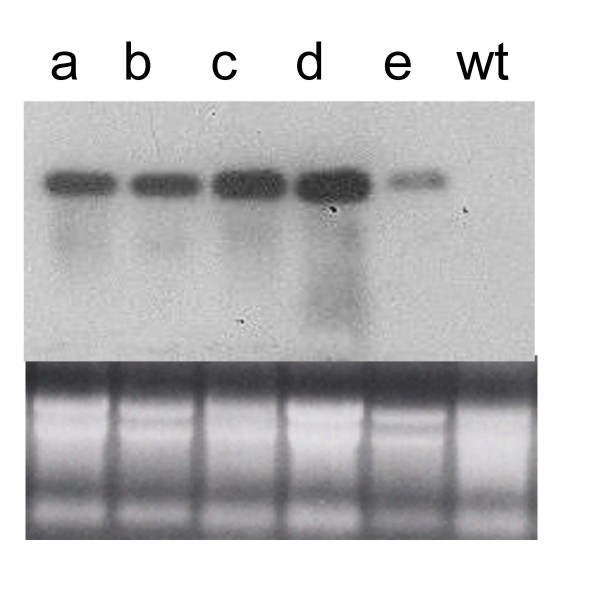
**Northern blot analysis of the recovered tomato lines expressing HVP16 L1 mRNA**. Total RNA was extracted from tomato fruits and 20 μg were fractionated by gel electrophoresis and processed as described in the legend to Fig. 1. The autoradiography (top panel) and the ethidium bromide stained gel (bottom panel) are shown. a and b, Transgenic lines L1. c, d and e, Transgenic lines L1-E6/E7. wt, non-transformed plant.

Total RNA isolated from wild type and transfected lines was used to determine L1 and L1-E6/E7 mRNA expression by northern blotting. As Fig. 2 shows, mRNA is accumulated to comparable levels in the two lines of L1 and in the two lines of L1-E6/E7. The latter displayed slightly higher accumulation that in L1 lines. One of the L1-E6/E7 lines, showed a band of lower intensity than the rest (Fig. 2).

**Figure 3:**
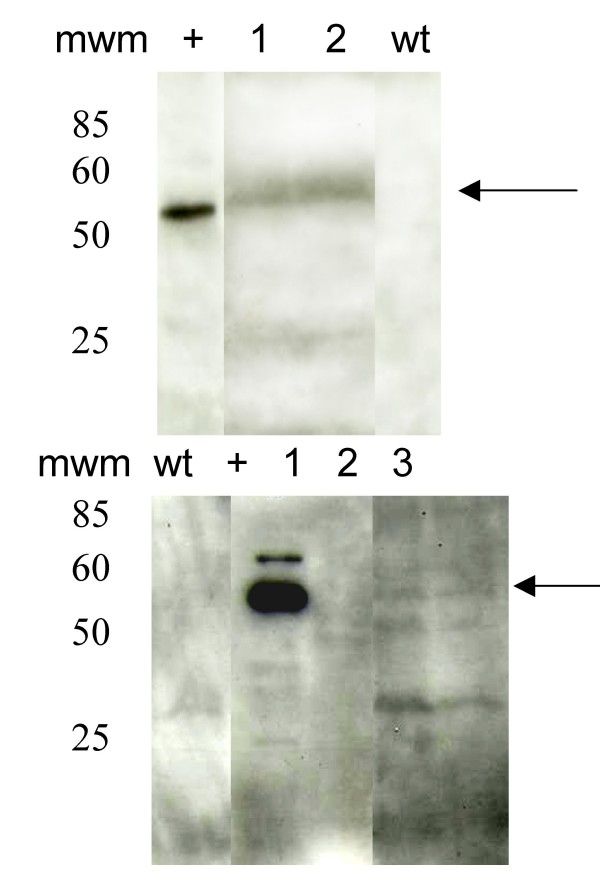
**Analysis of soluble protein extracts by immunodetection**. Total proteins were extracted from tomato fruit from the T1 generation and 40 μg were loaded per lane, separated by 12% SDS PAGE, transferred onto a PDVF membrane, incubated with a mouse monoclonal anti-L1 antibody and the bands detected by chemiluminescence as described in the text. Top panel, transgenic lines L1. Bottom panel, transgenic lines L1-E6/E7. mwm molecular weight markers, wt non-transformed tomato plant. The arrows indicate the bands present in transformed plants but not in non-transformed plants. Additional bands detected by the antibody can be seen in the bottom panel.

Western blot analysis showed a ≈55 kDa band present in fruit extracts from all lines and reacting with specific antibodies against L1 (Fig. 3). The band was not detectable in protein extracts from non-transformed fruit. Additional bands were also detectable by the antibodies in the case of lines transformed with L1-E6/E7. This could be due to processing of the recombinants proteins by an unknown protease or alternatives origins of translation at the L1 and L1E6/E7 sequences. One of the lines displayed a very faint band and it corresponds to the line yielding a band of low intensity in western blot. No signal was detected in untransformed plant sample.

The amount of L1 and L1-E6/E7 protein in tomato plant extracts was determined by ELISA at 405 nm using the commercial vaccine as standard. Yields of L1 protein harvested from tomato fruits were calculated to be between 0.05% to 0.1% of total soluble protein. When we determined by ELISA the expression of L1-E6/E7 in T2 plants from one of our lines, we found that the levels in the fruit were similar to the parent plant (data not shown), indicating that expression of the transgene is stable through generations. The lines presenting the highest expression levels were selected for further work.

### Electron microscopy

**Figure 4:**
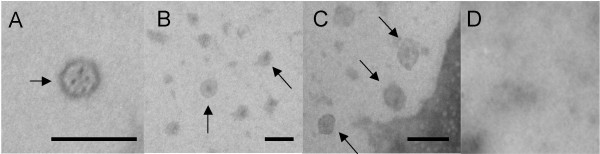
**VLP and cVLP structures formation**. Crude extracts of tomato fruit were processed as described in the text and negatively-stained with 2% phosphotungstic acid for 5 min. The samples were imaged on a Philips Morgagni 268 transmission electron microscope. The arrows indicate the putative VLPs observed. (**A**) Commercial vaccine Gardasil^® ^as control. (**B**) Transgenic lines with L1. (**C**) Transgenic lines with L1-E6/E7. (D) non-transformed plant. Bars indicate 0.1 μm.

L1 can self-assemble into VLPs in plant cells as noted before. We were interested to determine whether the plant-based, optimized L1 fused to the string of epitopes was able to assemble into chimeric VLPs. To this end we employed electron microscopy. As Figure 4 shows, VLPs of about 50–60 nm in diameter were observed in protein extracts obtained from tomato lines containing L1-E6/E7 which looked similar identical to LI particles. Protein extracts derived from non-transfected tomato fruits did not show any particles. Addition of the 50 amino acids containing the epitopes did not seem to affect assembly of the particles.

### Immunogenicity of plant-derived HPV-16 L1

**Figure 5:**
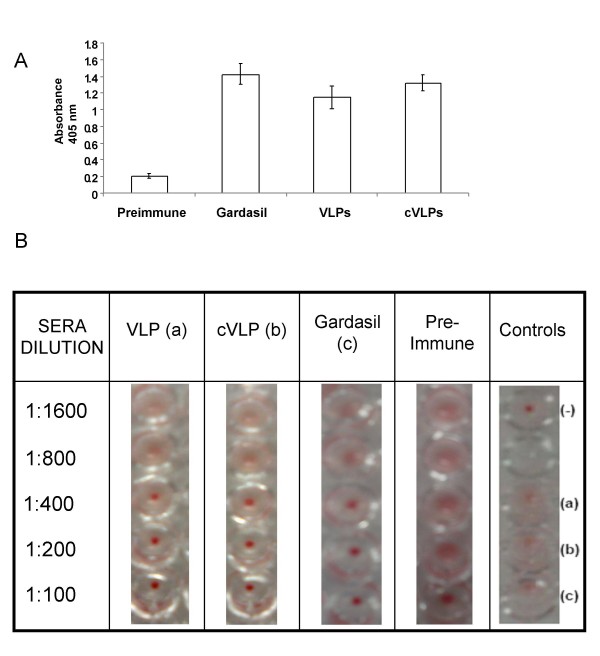
**Determination of the antibodies response of mice to VLPs by ELISA and hemmaglutination inhibition assay**. Groups of C57BL/6 mice were immunized with Gardasil^® ^and with purified VLPs and cVLPs derived from tomato fruits. Serum was collected from each mice and used for: A, determination of antibodies against different types of VLPs by ELISA. Bars, mean and SE (n = 6). Sera dilution 1:100. B, hemagglutination inhibition assay: sera from C57BL/6 mice (n = 6 in all cases) immunized with Gardasil^®^, VLPs and cVLPs and the pre-immune group were assayed at 1:1600, 1:800, 1:400, 1:200 and 1:100 dilutions as described in the text. (-), negative control (erythrocytes in the absence of VLPs), 100 ng of VLPs (a), cVLPs (b), and Gardasil^® ^(c). VLPs incubated with erythrocytes were used as positive controls for the hemagglutination assay.

It was important to determine if the plant-based particles could induce an HPV-16 L1 immune response and, secondly, if the E6/E7 epitopes displayed by the chimeras were immunogenic. Mice sera were collected 15 days after the last immunization and evaluated by ELISA. The results indicated that immunization with plant-based recombinant VLPs was associated with a significant serum antibody responses (Fig. 5A). The response was comparable to that obtained with an equivalent amount of commercial vaccine. Mice receiving only the adjuvant demonstrated no response at any time by ELISA.

To investigate whether the antibodies induced by the particles were neutralizing, we employed an indirect assay. The assay was based on the fact that VLPs interact with cell receptors present in the membranes of erythrocytes, resulting in agglutination [22]. Therefore we performed serial dilutions of mice sera and each dilution was incubated primarily with purified VLPs and subsequently with erythrocytes from mouse. As shown in figure (5B), antibodies from mice immunized with VLP, cVLP and commercial vaccine were able to inhibit hemagglutination completely from dilution 1:400. Once again, inhibition of hemagglutination of the plant-based VLPs was comparable to that of the commercial vaccine. In contrast, sera from mice immunized with adjuvant alone did not inhibit agglutination at any dilution (Fig. 5B).

### Cytotoxic activity of T cells obtained from mice immunized with VLPs

**Figure 6:**
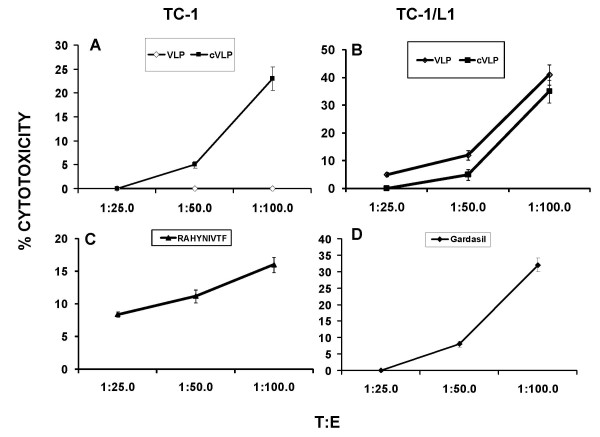
**Immunization with VLPs and cVLPs induce cytotoxic specific T-lymphocytes in C57BL/6 mice**. T lymphocytes derived from C57BL/6-immunized mice with Gardasil^® ^(n = 6), VLPs (n = 3) or cVLPs (n = 6), as well as with the HPV 16 E749-57 (RAHYNIVTF, H-2Db-restricted) epitope (n = 6), were restimulated in vitro with the epitopes HPV 16 E749-57 (RAHYNIVTF, H-2Db-restricted) and HPV 16 L1 165–173 (AGVDNRECI, H-2Db-restricted) and their cytolytic activity was tested by a standard 4-h ^51^Cr release assay against TC-1 cells A and C; and on HPV 16 L1-transfected TC-1 cells (TC-1/L1) B and D. T:E, target effector cells ratio.

In order to investigate the cellular immune response of mice immunized with plant-based VLPs, T-CD8+ cells were isolated from spleens and incubated in vitro with purified VLPs in the presence of IL-2. Subsequently, CTL activity was determined on a tumor epithelial cell line from mouse lung co-transfected with E6 and E7 (from HPV 16) and c-Ha-ras, denominated TC-1 [23] as well as on TC-1 cells expressing the L1 HPV 16 gene (TC-1/L1). As Fig. 6A shows, only T-CD8+ cells derived from mice immunized with cVLPs were able to lyse TC-1 cells, whereas T-CD8+ cells from mice immunized with VLP did not present this activity. However, when TC-1/L1 cells were employed, both cVLPs and VLPs were able to lise TC-1 cells (Fig. 6B). Similar results were obtained when T-CD8+ cells from mice immunized with commercial vaccine were incubated with TC-1/L1 cells (Fig. 6D). These results suggested that immunization with both types of plant-based VLPs elicit the generation of T cells specific to epitopes from L1 or from E6/E7 insert. To investigate further this possibility, C57BL/6 mice were immunized with the epitope RAHYNIVTF derived from E7, which is included in the cVLPs. As can be observed in Fig. 6C, T-CD8+ cells obtained from the spleen of these mice were also able to lyse TC-1 cells, which confirms that CTL activity of cVLPs is conferred by the string of epitopes.

## Discussion

In this work we report for the first time the expression of L1-based chimeric VLPs from HPV-16 containing a string of epitopes from E6 and E7 HPV-16 proteins. There have been a few instances of chimeric VLPs produced in other systems (baculovirus, yeasts, *E. coli*) but not in plants.

Only a low number of transgenic plants containing the L1 constructs could be recovered, suggesting that L1 expression may interfere with plant growth and viability. Similar results had been obtained previously in potato expressing L1 [14]. Since expression of native or plant codon optimized L1 was problematic in plants, resulting in low expression levels, we had decided to employ the optimized L1 cDNA reported by Collier et al. [21], since the highest expression in plants had involved a codon-optimized gene [11]. The levels of expression obtained in this work (0.05% – 0.1% TSP) were higher or comparable to what had been reported in previous works, 23 ng/g, 0.2% TSP and .034–.076% TSP in potato and tobacco [7,10,14], but they are not comparable to what Maclean et al. [11] obtained using chloroplast transformation (11% TSP). Nevertheless, we were still able to purify VLPs in sufficient amounts to perform the immunization experiments.

HPV L1 VLPs can tolerate foreign peptides within the surface-exposed areas. Regions of the C terminus can be deleted and replaced with heterologous amino acids without loss of VLP structure. Muller et al. [24] removed the last 34 amino acids from the C terminus of HPV 16 L1 and replaced them with an E7 fragment of 50 amino acids and the VLPs could still assemble. Furthermore, it had been reported that up to 60 amino acids could be cloned fused to the C-terminus of L1, and VLPs would still be formed [25]. We removed the last 34 amino acids from the C terminus, inserting 50 amino acids and the particles assembled adequately. Analysis of the tomato lines obtained showed that they contained only one copy of the transgene, whereas the mRNA of VLPs and cVLPs accumulated to high levels in fruits of all lines. A band displaying the same size as that obtained from the commercial vaccine was detectable by specific antibodies in fruit from all lines indicating successful expression in tomato. The plant-produced VLPs were able to induce neutralizing antibodies in mice immunized intraperitonally as demonstrated by a hemagglutinin inhibition assay. Previously L1-based VLPs alone had been produced in plants and demonstrated to be immunogenic in mice when administered either orally or intraperitonally [7,8,14]. Our results are confirmatory of those findings but it was important to demonstrate that our chimeric particles also possess prophylactic as well as therapeutic properties (see below). Interestingly, the E6 T-cell epitope employed in this work, had not been included in cVLPs before.

Since we are interested in producing a prophylactic/therapeutic preparation it was important to investigate whether the presence of the string of epitopes could elicit any T cell cytotoxic activity. Previously, Castellanos et al [26] had tested a series of different epitopes from E6 and E7 HPV-18 and found that only two of them (from E7) were able to induce cell lysis in vitro. Our approach is different in that we combined a prophylactic/therapeutic preparation. The epitopes employed in this work had been reported as tumor rejection antigens, causing regression of disease and resolution of viral infection [17-19]. Our results indicate that immunization with plant-based cVLPs elicits the generation of CTL activity against the epitopes present in the particle. This conclusion is supported by the results of immunization with the E7 epitope RAHYNIVTF alone which yielded similar results to those obtained with immunization with cVLPS. VLPs alone or the commercial vaccine did not present this activity. These results are in agreement with previous experiments using cVLPs prepared in baculovirus or *E. coli*. For example, Schafer et al. [25] inserted 60 N-terminal amino acids of the HPV-16 E7 protein into the C-terminus of L1. These chimeric particles induced conformational antibodies against L1 and a robust CTL response against E7 in mice. Kuck et al. [27] produced in baculovirus HPV 16 cVLPs containing L1 fused to the first 60 amino acids from E7 and were able to not only induce L1-specific antibodies but also L1- and E7-specific CTL responses after DNA vaccination. Bian et al. [23] using a similar approach, produced in *E. coli *HPV-16 capsomers containing the first 60 amino acids from E7. These capsomeres were able to induce conformational and neutralizing antibodies against HPV virus-like particles and trigger cell-mediated specific immune responses against the L1 and E7 peptides. Freyschmidt et al. [28] prepared HPV 16 cVLPs L1-E7 fusion proteins and in preclinical studies the cVLPs were shown to induce neutralizing antibodies and L1- and E7-specific T cell responses. Chimeric VLPs with L1 and E7 have been employed in clinical trials but there was a poor match between the clinical response and the immune response induced within the individual patients [for a review see [29]]. For that reason, Freyschmidt et al. [28] emphasized the need of improved immunogenicity in future clinical trials and to that end they employed lipopolysaccharides, unmethylated CpG motifs and sorbitol, which substantially increased the immunogenicity of cVLPs.

Chimeric VLPs have also been prepared by fusing L1 to HPV genes such as L2, E1 and E2. However, the use of these genes for therapeutic purposes is still somewhat controversial because the T cell response to them is not too well defined [30,31]. In this work, we decided to employ well defined T-cell epitopes from E6 and E7 mainly because of their proven effects and because the immune response to some of these epitopes had been reported not to be HPV type-specific [17-19]. This approach presents the additional advantage that the length of the fused insert to L1 can be kept within the minimum necessary to allow formation of VLPs by a judicious choice of epitopes. The order of the epitopes seems to be important. A specific order of epitopes may have dramatic may effects and altering that order may boost or diminish the response [32,33]. It remains to be determined whether the CTL response can be boosted by altering the order of our epitopes. HPV VLPs have been postulated as ideal vehicles to deliver epitopes or genes and for that reason a wide range of foreign peptides have been fused and expressed as chimeric VLPs in different systems or administered as DNA [20]. Many of this cVLPS have shown antitumoral activity [29]. Cellular immune responses are essential in controlling cervical cancer and in most cases cVLPs are able to fully activate dendritic cells, and are subsequently capable of inducing endogenously processed, epitope-specific human T-cell responses in vitro [34,35]. Dendritic cells are the most potent inducers of immune responses and play a central role in VLP-induced immunity. Hence, cVLPs may prove fundamental for the development of therapeutic vaccination.

An ideal HPV vaccine should be prophylactic as well as therapeutic. Our results demonstrate for the first time the production in plants of chimeric VLPs containing a L1-based VLPs fused to a string of epitopes from E6 and E7 proteins, which were able to elicit humoral and CTL activity. The use of plants for production of a cost-effective, efficient HPV vaccine remains a very attractive possibility, currently investigated by several groups, including ours. As Freyschmidt et al. [28] indicate, inclusion of an appropriate adjuvant may alleviate the problem of low immunogenicity.

## Conclusion

We report for the first time the expression in plants of a HPV 16 L1-based cVLPs containing a string of T-cell epitopes from HPV 16 E6 and E7 fused to the C-terminus. The chimeric particles were able to induce neutralizing antibody and a significant cytotoxic T-lymphocytes activity. Experiments in vivo are in progress to determine whether the chimeric particles are able to induce regression of disease and resolution of viral infection in mice. Chimeric particles of the type described in this work may potentially be the basis for developing prophylactic/therapeutic vaccines. The fact that they are produced in plants, may lower production costs considerably.

## Materials and methods

### Construction of the chimeric VLP and tomato transfection

**Figure 7:**
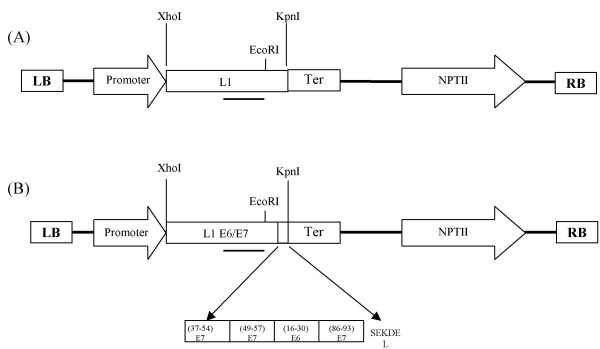
**Expression vectors**. Map showing the Ti region of constructs (**A**) L1 and (**B**) L1-E6/E7. The promoter and the terminator (Ter) employed were from the Cawliflower Mosaic Virus. The bold line represents the EcoRI/BamH1 fragment (670 bp) that was used as probe for both Southern and northern blot analysis. LB: Left border, RF: Right border.

An HPV-16 L1 sequence in which inhibitory sequences had been modified at the 5' end [21] was amplified by PCR using specific primers and cloned into pCAMBIA 2301 between the promoter and terminator of 35S CaMV. The forward primer was: 5'-CCGGAATTCATGAGCCTGTGGC-3' and the reverse primer was: 5'-ATTGGGTACCTTACAGCTTACG-3'. We synthesized a sequence containing a string of epitopes mediating T-cell cytotoxic activity from the E6 and E7 HPV-16 genes [17-19]. The synthesis was performed by GenScript . The string of epitopes (EIDGPAGQAEPDRAHYNIVTFPARKLPQLCTELQTTITLGIVCPISEKDEL) included amino acids 16–30 from E6 and 37–54, 49–57 and 86–93 from E7, ending with the signal for retention in the endoplasmic reticulum, SEKDEL. This sequence was fused in-frame at the 3' end of the L1 HPV-16 gene by replacing the 34-carboxy-terminal amino acids of the structural protein by PCR as described by Muller et al. [24], to generate the chimerical construct LI-E6/E7 (Fig. 7). The construct was verified by sequencing and inserted between the promoter and polyadenylation signal of CaMV 35S, between the XhoI/KpnI restriction sites. The whole plant expression cassette was inserted into the binary Ti plasmid pCAMBIA 2300 to obtain the LI-E6/E7-2300 construct, which was transferred by electroporation into *Agrobacterium tumefaciens *strain LBA4404, and used for transformation of tomato cotyledons (*Lycopersicon esculentum *Mill).

Tomato plants transfection has been described in detail previously [36]. We employed the TA234 genotype kindly donated by Prof. Steve Tanksley from Cornell University. Putative transfected plants selected by kanamycin resistance were transferred to soil and grown in the greenhouse. Seeds from selected plants were collected, surface sterilized with 20% commercial blanch and 0.1% Tween 20 for 15 min and germinated on MS medium supplemented with 100 mg/1 kanamycin. Seeds from T0 transgenic plants were surface sterilized and germinated on rooting medium with 100 mg/l kanamycin. Transfected plantlets were grown in the greenhouse and utilized for further experiments. Integration of LI-E6/E7 HPV-16 fusion gene and chimeric protein in tomato plants was confirmed by Southern blot hybridization and expression was confirmed by northern and western blot analysis.

### Southern and northern blot analysis

For Southern analysis, genomic DNA was isolated from young leaves as described by Gutiérrez Ortega et al. [36]. Twenty micrograms of DNA were digested with EcoRI, which cuts only one in the gene, resolved by agarose electrophoresis and blotted on to Hybond-N^+ ^nylon membrane (Amersham Pharmacia Biotech) as described by Sambrook et al. [37]. The membrane was hybridized to a radioactively labeled probe synthesized with the Rediprime II kit (Amersham Pharmacia Biotech) using as template a 670 bp EcoRI/BamH1 fragment of LI-E6/E7 HPV-16 gene (Fig. 7). Hybridization was performed in the Rapid-Hyb buffer (Amersham Pharmacia Biotech) and the membrane was exposed to Kodak Biomax Film (Kodak) at -70°C. For northern analysis, total RNA was extracted from fruit with the Concert Plant RNA Reagent (Invitrogen) and 20 μg fractionated by denaturing formaldehyde gel electrophoresis [37]. Blotting, hybridization and exposure were done as described above. The probe was the same as that used for Southern analysis.

### Sample preparation and western blot analysis

Tomato fruits were ground in liquid nitrogen and homogenized in 4 volumes of ice-cold extraction buffer (0.01 M Na_2_HPO_4_, 0.002M KH_2_PO_4_, 0.137 M NaCl, 0.027 M KCL and 0.025 M sodium ascorbate) in the presence of 1 mM phenylmethylsulphonyl fluoride (PMSF) or protease inhibitors cocktail (Sigma-Aldrich). Cleared supernatants were collected after centrifugation at 13,000 rpm for 10 min at 4°C and employed for western blot analysis and LI-E6/E7 HPV-16 determination by ELISA. Forty micrograms of total soluble protein aliquots, as determined by Bradford [38], were resuspended in loading buffer (0.1 M Tris pH 8.0, 2% SDS and 0.75 M β-mercaptoethanol), boiled for 5 min, resolved by 12% SDS-PAGE and electrotransferred on to polyvinylidene difluoride (PVDF) membrane. The membrane was subsequently blocked with 8% non-fat milk, 0.1% Tween-20 in TBS for 1 h at room temperature. Mouse monoclonal anti-L1HPV 16 (Chemicon) at 1:500 and HRP conjugated goat anti mouse IgG (Amersham Pharmacia Biotech) at 1:10,000 were used for L1 and LI-E6/E7 HPV 16 detection, which was carried out by chemiluminescence (ECL Reagent, Amersham Pharmacia Biotech). The commercial HPV vaccine Gardasil^® ^was used as positive control.

ELISA plates (Corning) were coated with 100 μl of crude extract of tomato fruits or chromatography-purified VLPs and cVLPs. Gardasil^® ^was used as standard. All samples were tested in triplicate. Plates were incubated for 16 h at 4°C, washed twice in TBS-0.1% Tween 20 and blocked by addition of 2%w/v bovine serum albumin in TBS-0.1% Tween 20 with 2 h incubation at 37°C. Plates were washed four times, and incubated with sera at 1:500 in blocking buffer from women vaccinated with three doses of commercial vaccine. After 2 h at 37°C, plates were washed 6 times and incubated with 1:5000 rabbit-anti human IgG (Zymed, USA). The plates were incubated for a further 2 h at 37°C. After eight washes, alkaline phosphatase substrate (Sigma) was diluted in a 10% diethanolamine solution (pH 9.8) and added to the plates. The absorbance (405 nm) was determined in an ELISA plate reader. The concentration of VLPs was calculated by average of three repeats.

### Electron microscopy

Non-transfected and tranfected L1 fruits were ground under liquid nitrogen. The resulting powder was suspended in 5 volumes of extraction buffer PBS, pH 7.2, in the presence of the protease inhibitor cocktail (Sigma) and kept on ice. The suspension was clarified by centrifugation (30 min at 1,500 × *g*, followed by 30 min at 10,000 × *g*). The clarified supernatants were then centrifuged at high speed (3.5 h at 100,000 × *g*), and the final pellets were resuspended in 1 ml of PBS. Twenty microliters of this suspension were mixed with an equal volume of 2% phosphotungstic acid for 5 min. The mix was placed on Formvar grids for 30 min and excess liquid absorbed with a soft tissue. The grids were imaged on a Philips Morgagni 268 transmission electron microscope. Commercial vaccine at 1:50 dilution in PBS was used as positive control.

### VLP and cVLP purification

Tomatoes from HPV 16 L1 and HPV 16 L1 E6/E7 transgenic plants, were lysed with extraction buffer (PBS pH 7.4 with 1 mM PMSF) on ice. The homogenates were clarified by centrifugation at 10,000 rpm for 30 min at 4°C and filtered on Whatman paper No. 1 and then on Millipore 0.8 μM filter. The supernatant was then applied on to an affinity column, prepared by coupling sera from women vaccinated with three doses of the commercial vaccine to 4B CNBr-Sepharose (Sigma). The columns were washed with 50 vol of washing buffer (50 mM Tris-HCl, 5 mM EDTA, 150 mM NaCl, 0.1% NP-40, pH 7.4), followed by 50 vols of the same buffer without NP-40. VLPs were eluted with 100 mM HCl-glycine, pH 4.0. The VLP-containing fractions were neutralized with 100 m*M *Tris-HCl (pH 9.0) and dialyzed against PBS overnight. The amount of plant-purified VLP/cVLP in fractions was measured by ELISA as previously described, using commercial vaccine as standard.

### Immunization of mice

Groups of 6 eight week-old C57BL/6 mice (H2-Db) (Harlan) were immunized as follows: one group was immunized intraperitonally and boosted 2 weeks later with 5 μg of VLPs alone in the presence of complete Freund's adyuvant (CFA), another with cVLPs and CFA and a third group with 5 μg of commercial vaccine (Gardasil^®^). Another group was immunized with 2 doses of 100 μg HPV 16 E749-57 (RAHYNIVTF, H-2Db-restricted) peptide (Invitrogen) and the other one received CFA according to the same schedule. Two weeks after the booster immunization, blood was obtained from each mouse and used to determine serum antibody titers. The mice were then sacrificed to remove the spleen to obtain T lymphocytes and then used for cytotoxic assays.

### ELISA for detecting anti-VLP antibodies

To determine the specific antibody titers in VLPs (Gardasil^®^, HPV 16 L1 and HPV 16 L1E6/E7)-immunized mice, 100 μl of Gardasil-derived VLPs at 1000 ng/ml diluted in PBS were used to coat 96-well ELISA plates for 16 h at 4°C. Plates were then washed four times with TBS containing 0.1% Tween 20 (TBS/Tween 20). Non-specific binding sites were blocked with 200 μl 2% BSA in TBS/Tween 20 for 2 h at 37°C. After washing, 100 μl serum samples (diluted from 1:100) were added in triplicate, and the plates were incubated at 37°C for a further 2 h. After washing, alkaline phosphatase-conjugated goat anti-mouse IgG (Zymed, USA) were diluted 1:500 in blocking buffer and 100 μl was added to each well. The plates were incubated for 2 h at 37°C. After washing, alkaline phosphatase substrate Sigma 104 was diluted in a 10% diethanolamine solution (pH 9.8) and added to the plates. The absorbance (405 nm) was determined in an ELISA plate reader. The concentration of VLPs was calculated by average of three repeats. The assay was considered valid only when the coefficient of variation of the triplicates was ≤10%. Additionally, all samples were tested on two wells not coated with peptide to define non-specific reactivity. The final ELISA value was calculated by subtracting the non-specific reactivity mean absorbance from the triplicate mean absorbance.

### Hemagglutination inhibition assay

Hemagglutination inhibition assays were performed according to Roden et al. [22]. Mouse blood was collected in a heparinized tube and erythrocytes separated by centrifugation at 1,000 rpm for 5 min at 4°C, washed twice with PBS and suspended in dilution buffer (1% BSA in PBS) at a concentration of 1% (v/v). Purified VLPs (100 ng) were then incubated with various dilutions of sera samples at room temperature for 2 h, after which the samples were diluted with an equal volume of the 1% RBC suspension. Aliquots (100 μl) of the mixtures were transferred to a round-bottomed, 96-well plate and incubated for 3 h at 4°C. Spots indicating VLP neutralizing activity were photographed.

### T-cell stimulation and cytotoxicity assays

Primed T cells from spleen of VLP-immunized mice were re-stimulated using antigenic peptides [23]: HPV 16 E749-57 (RAHYNIVTF, H-2Db-restricted) and HPV 16 L1 165–173 peptide (AGVDNRECI, H-2Db-restricted) or 1 μg commercial vaccine, recombinant, purified VLPs and 100 U/mL of mrIL-2 (R&D Systems). TC-1 cells derived from a C57BL/6 lung tumor epithelial cells co-transformed with HPV 16 E6/E7 and c-Ha-ras [39] as well as TC-1 transfected with HPV 16 L1 gene (TC-1/L1) were used as targets after labeling with ^51^Cr (Amersham) for 1 h. Different numbers of effector cells obtained from VLP-immunized mice in 50 μL of complete medium were incubated and then 1× 10^451^Cr-labeled, target cells were added to triplicate wells of 96-well plates in a final volume of 200 μL. After 4 h at 37°C, 100 μl of supernatant were measured on a γ-counter (Packard). For each pretreated cell group, ^51^Cr labeled cells incubated with 5% Triton X-100 or medium alone were used to determine maximum and spontaneous releases. Spontaneous release was usually less than 10% and never exceeded 15%. The percentage of specific lysis of each well was calculated as: (experimental release - spontaneous release)/(maximal release - spontaneous release) × 100.

## Competing interests

The authors declare that they have no competing interests.

## Authors' contributions

GPR carried out the vector construction, transfection and culture of tomato, molecular studies of the plants and drafted the manuscript. AMG designed the immunological studies and performed cytotoxicity assays. MLMG participated in the immunization, collection and storing of sera samples from women vaccinated with Gardasil^®^. CGRP performed the electron microscopy analyses. JHM performed the T lymphocyte activation assays. BWS participated in discussion of results and revision of the manuscript. MAGL conceived of the study, participated in its design and coordination and wrote the manuscript. All authors read and approved the final manuscript.
